# Supplementary figures and images for: Hiding in plain sight: New virus genomes discovered via a systematic analysis of fungal public transcriptomes
Source: PLoS One. 2019 Jul 24;14(7):e0219207. doi: 10.1371/journal.pone.0219207 (PMC6655640; doi:10.1371/journal.pone.0219207)

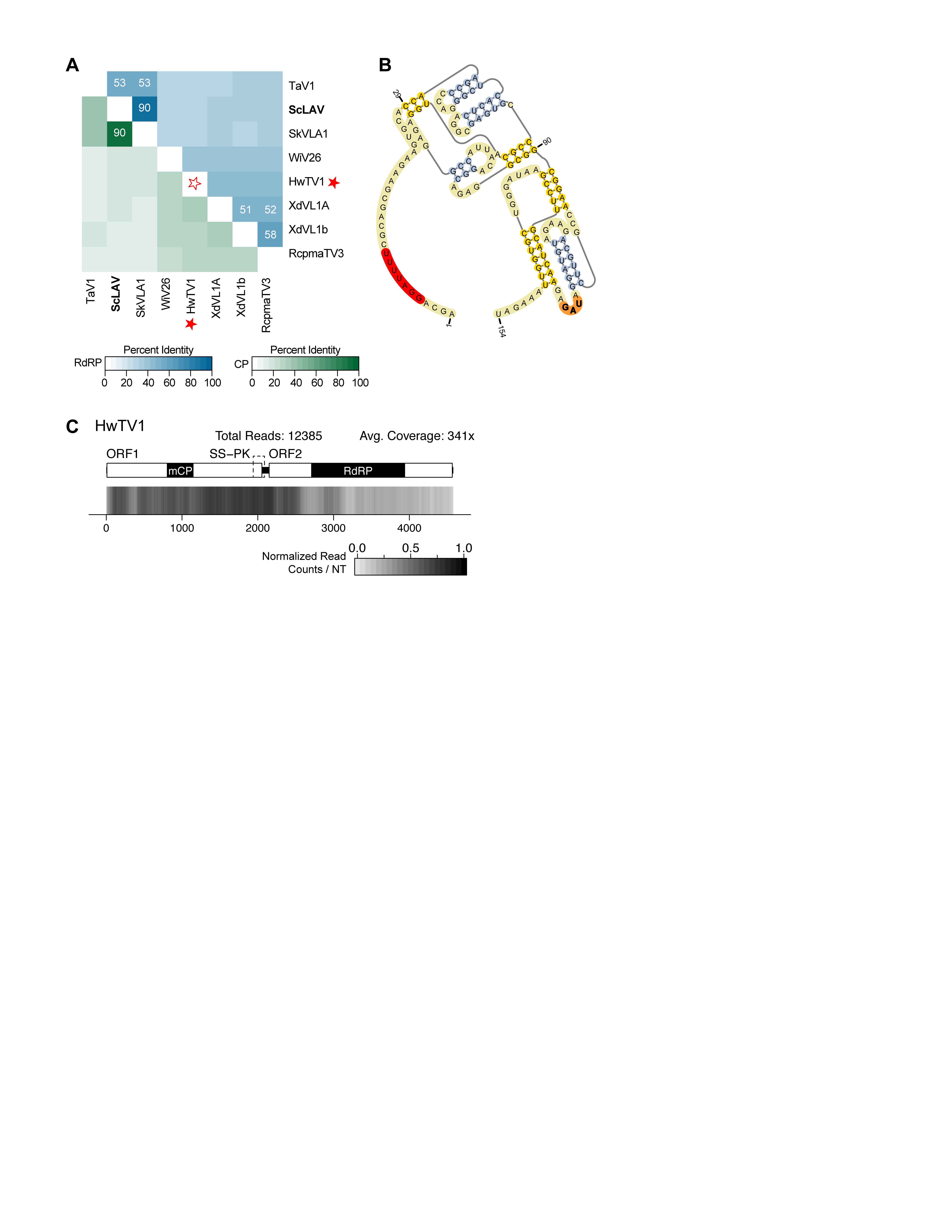

Supplement: S1 Fig — (A) Percent identity matrix, generated by Clustal-Omega 2.1, to select totiviruses. ICTV-recognized members of the genus are in bold. The top half of the matrix, in the blue scale, is the percent identity of RdRP protein sequences and the bottom half of the matrix, in the green scale, is the percent identity of CP protein sequences. For sake of clarity, the 100% identity along the diagonal has been removed and outlined stars are used to note junction point for the row and column of the virus identified. Identities ≥50% are noted. Full names and accession numbers for protein sequences are in S2 Table. (B) Slippery site and two pseudoknot structures predicted using DotKnot and visualized with PseudoViewer3. The putative slippery site heptamer (GGAUUUU) is highlighted in red and the CP stop codon (UAG) is in highlighted in orange. (C) Density plot of read counts per nucleotide across the genome of HwTV1. Graphical depiction of the genome structure, encoded genes and predicted protein motifs are on top and a heatmap of normalized read counts is on the bottom. The predicted slippery site and pseudoknot region is indicated by the dashed box. Read counts are normalized to a 0–1 scale, where the maximum read count for the genome equals 1. Total reads mapped to the virus genome and average read coverage are noted. Viruses identified in this study are noted with a red star. RdRP: RNA-dependent RNA Polymerase; CP: Coat Protein; mCP: major coat protein domain of L-A virus. (TIF) [file pone.0219207.s001.tif]

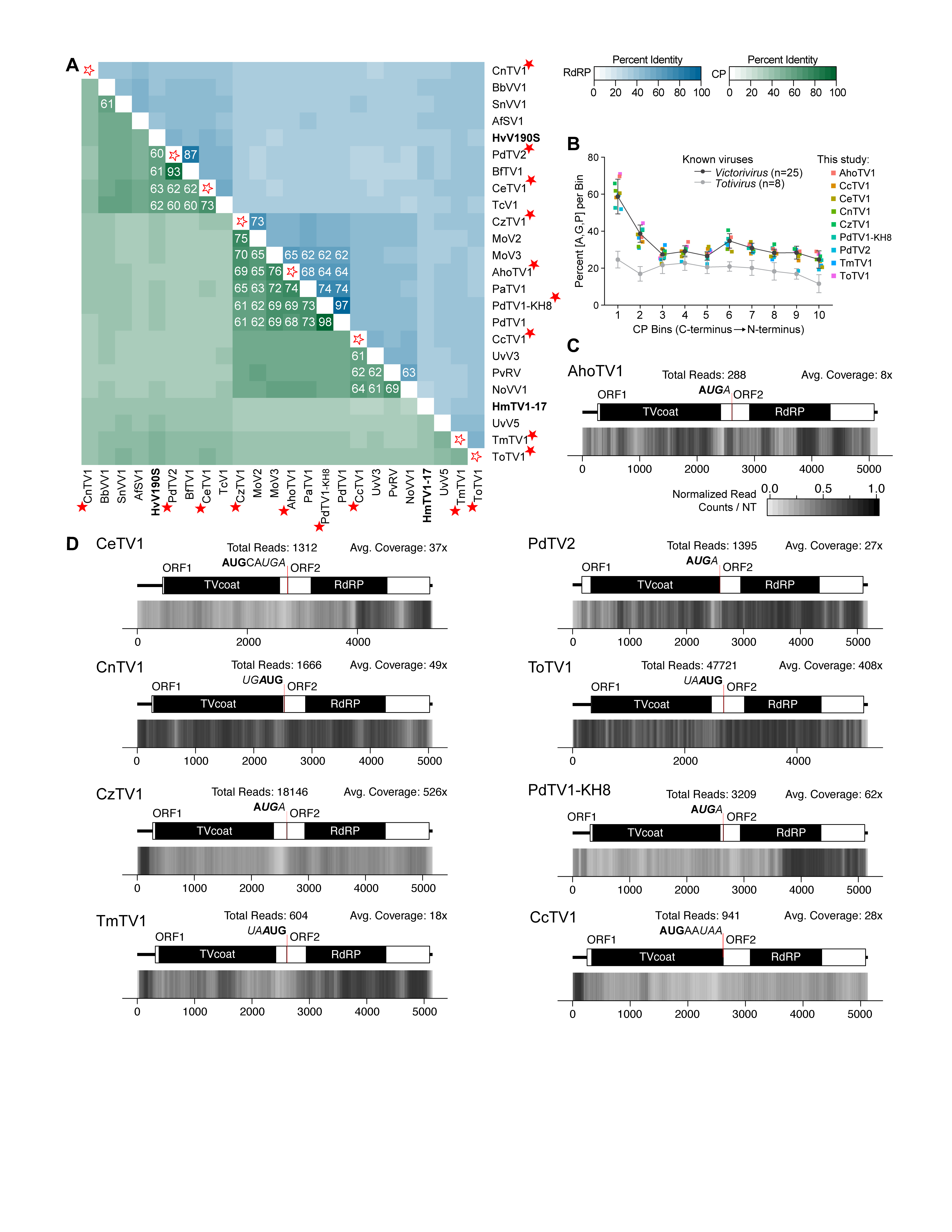

Supplement: S2 Fig — (A) Percent identity matrix, generated by Clustal-Omega 2.1, comparing identified viral sequences to select members of the genus Victorivirus. The top half of the matrix, in the blue scale, is the percent identity of RdRP protein sequences and the bottom half of the matrix, in the green scale, is the percent identity of CP protein sequences. For sake of clarity, the 100% identity along the diagonal has been removed and outlined stars are used to note junction point for the row and column of the viruses identified. Where the identified viral sequences have significant similarity to known viral sequences is indicated, specifically RdRP or CP sequences with similarity ≥60%. Virus names in bold are ICTV-recognized species within the genus. Full names and accession numbers for protein sequences are in S2 Table. (B) Percent of alanine, glycine, and proline (A, G, P) amino acids in the CP coding sequence. The binned values were calculated for twenty-five known victoriviruses and ten known totiviruses, shown as dark and light grey lines respectively. The nine putative victoriviruses identified in this study were individually analyzed and plotted. (C and D) Density plot of read counts per nucleotide across the viral genome for the nine viruses identified. Graphical depiction of the genome structure, predicted ORFs, and putative motifs are illustrated on top and a heatmap of normalized read counts is on the bottom. Reads are normalized to a 0–1 scale, where the maximum read count for a given genome equals 1. Total reads mapped to the virus genome and average read coverage are noted for each virus. The overlap between the CP stop codon (italics) and RdRP start codon (bold) is also indicated. Red stars indicate viruses identified in this study. RdRP: RNA-dependent RNA Polymerase; CP: Coat Protein; ORF: Open Reading Frame. (TIF) [file pone.0219207.s002.tif]

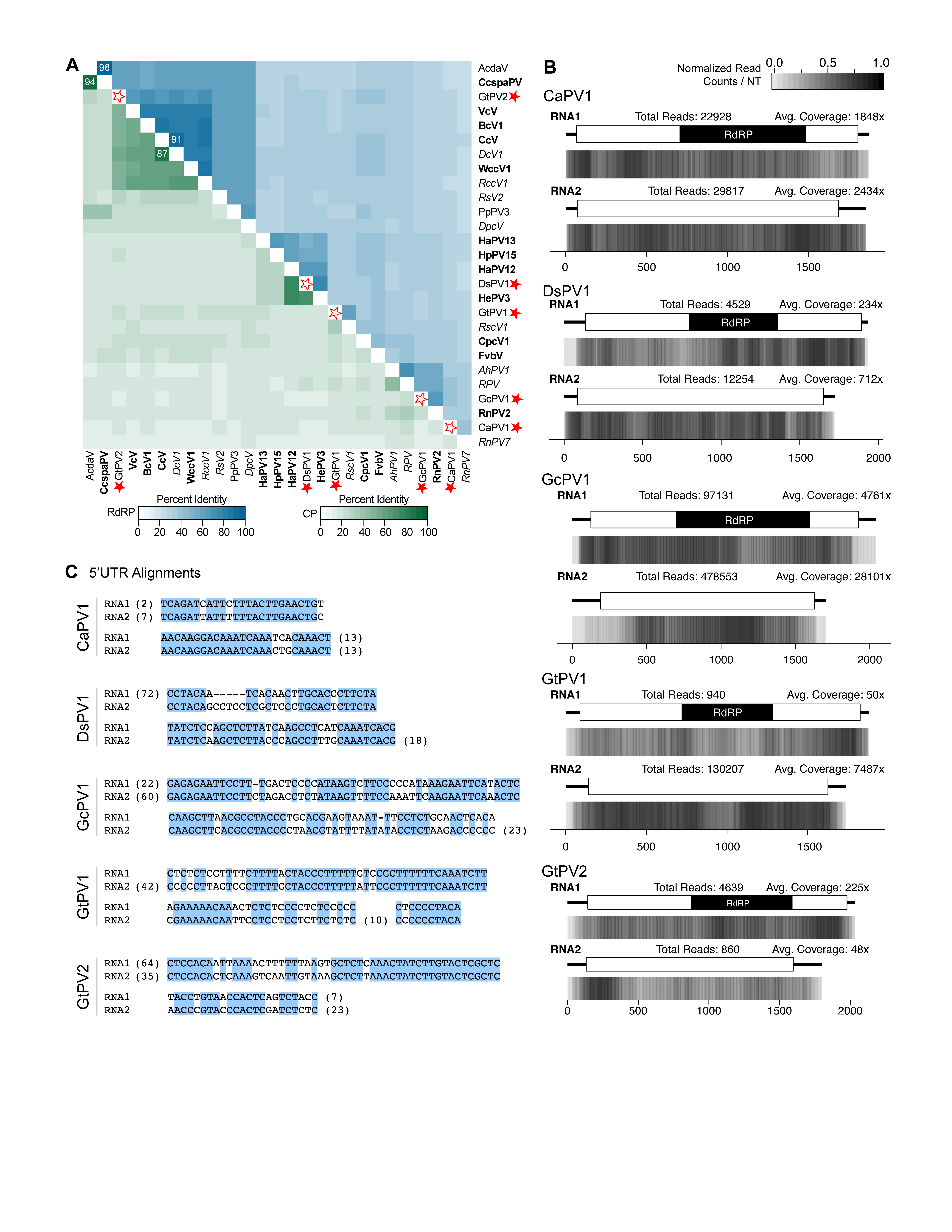

Supplement: S3 Fig — (A) Percent identity matrix generated by Clustal-Omega 2.1 of viruses identified in this study and select alphapartitiviruses. The top half of the matrix, in the blue scale, is the percent identity of RdRP protein sequences and the bottom half of the matrix, in the green scale, is the percent identity of CP protein sequences. For sake of clarity, the 100% identity along the diagonal has been removed and outlined stars are used to note the junction for the row and column of the viruses identified. Identities above the species demarcation are noted: ≥90% for RdRP and >80% for CP. Names in bold are ICTV-member species, while names in italics are related, unclassified viruses. Full names and accession numbers for protein sequences are in S2 Table. (B) Density plot of read counts per nucleotide across the viral genome for the five alphapartitiviruses identified in this study. Graphical depiction of the genome structures of RNA1 and RNA2, the predicted ORFs, and RdRP motif are on illustrated above the heatmap of normalized read counts. Reads are normalized to a 0–1 scale, where the maximum read count for a given genome equals 1. Total reads mapped to the virus genome and average read coverage are noted for each virus. (C) Alignment of 5’UTRs of RNA1 and RNA2 genome segments for each virus. Residues not shown in alignment are in parentheses. Blue shading indicates regions of 100% identity. Red stars note the viruses identified in this study. RdRP: RNA-dependent RNA Polymerase; CP: Coat Protein. (TIF) [file pone.0219207.s003.tif]

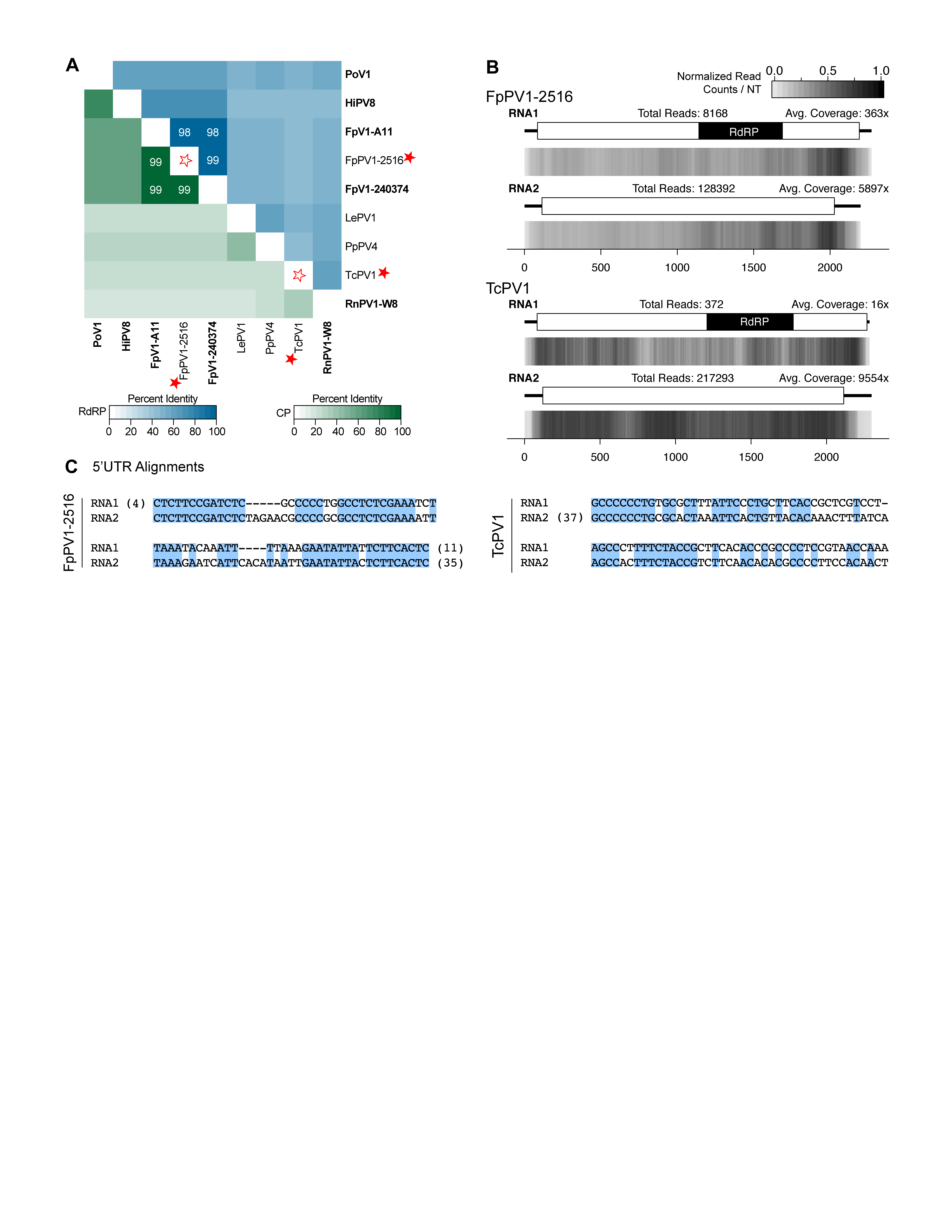

Supplement: S4 Fig — (A) Percent identity matrix, generated by Clustal-Omega 2.1, between identified viruses and select betapartitiviruses. The top half of the matrix, in the blue scale, is the percent identity of RdRP protein sequences and the bottom half of the matrix, in the green scale, is the percent identity of CP protein sequences. For sake of clarity, the 100% identity along the diagonal has been removed and outlined stars are used to note junction point for the row and column of the viruses identified. Where the percent similarity is above the cutoffs for species demarcation is noted, specifically >90% for RdRP sequences and >80% for CP sequences. Bolded names are ICTV-recognized member species. Full names and accession numbers for protein sequences are in S2 Table. (B) Density plot of read counts per nucleotide across the viral genome for the two Betapartitiviruses. Graphical depiction of the genome structure of the two RNA genome segments, the predicted ORFs, and the RdRP motif are along the top and a heatmap of normalized read counts is on the bottom. Read counts are normalized to a 0–1 scale, where the maximum read count for a given genome equals 1. Total reads mapped to the virus genome and average read coverage are noted for each virus. (C) Alignment of 5’UTR sequences from the RNA1 and RNA2 genome segments for both viruses. Blue boxes highlight conserved nucleotides and numbers in parentheses are residues not shown. Viruses identified in this study are noted with a red star. RdRP: RNA-dependent RNA Polymerase; CP: Coat Protein. (TIF) [file pone.0219207.s004.tif]

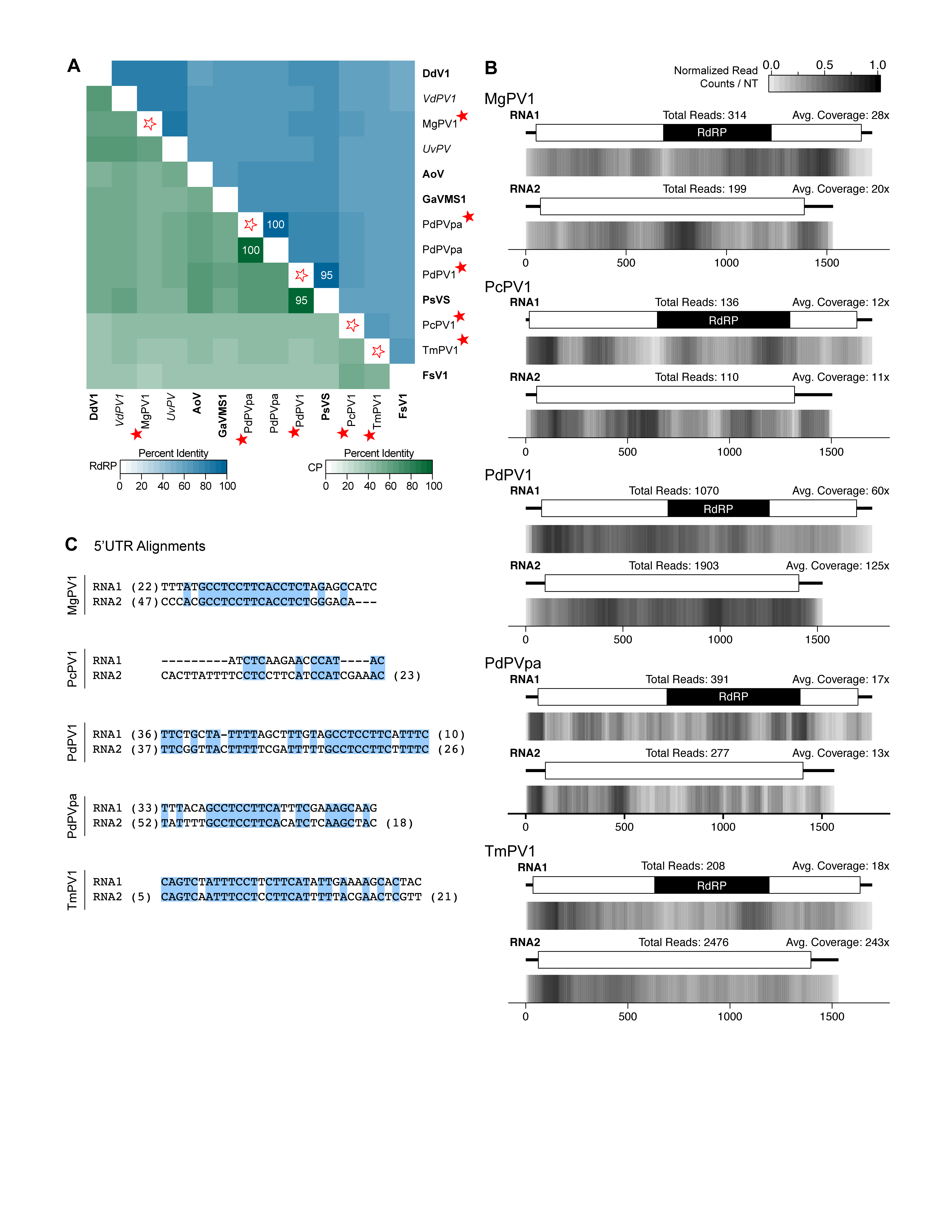

Supplement: S5 Fig — (A) Percent identity matrix, generated by Clustal-Omega 2.1, between identified viruses and select gammapartitiviruses. The top half of the matrix, in the blue scale, is the percent identity of RdRP protein sequences and the bottom half of the matrix, in the green scale, is the percent identity of CP protein sequences. For sake of clarity, the 100% identity along the diagonal has been removed and outlined stars are used to note junction point for the row and column of the viruses identified. Where sequences have a value greater than the species demarcation cutoff is noted, specifically >90% for RdRP and >80% for CP sequences. Bolded names are ICTV-recognized member species while italicized names are related, but unclassified viruses. Full names and accession numbers for protein sequences are in S2 Table. (B) Density plot of read counts per nucleotide across the viral genome for the five gammapartitiviruses. Graphical depiction of the genome structure of the RNA segments, predicted ORFs, and RdRP motif are on top and a heatmap of normalized read counts is on the bottom. Read counts are normalized to a 0–1 scale, where the maximum read count for a given genome equals 1. Total reads mapped to the virus genome and average read coverage are noted for each virus. (C) Alignment of 5’UTR sequences of RNA1 and RNA2 for the identified viral sequences. Conserved nucleotides are highlighted with a blue box and number of nucleotides not shown in the alignment are noted in parentheses. Viruses identified in this study are noted with a red star. RdRP: RNA-dependent RNA Polymerase; CP: Coat Protein. (TIF) [file pone.0219207.s005.tif]

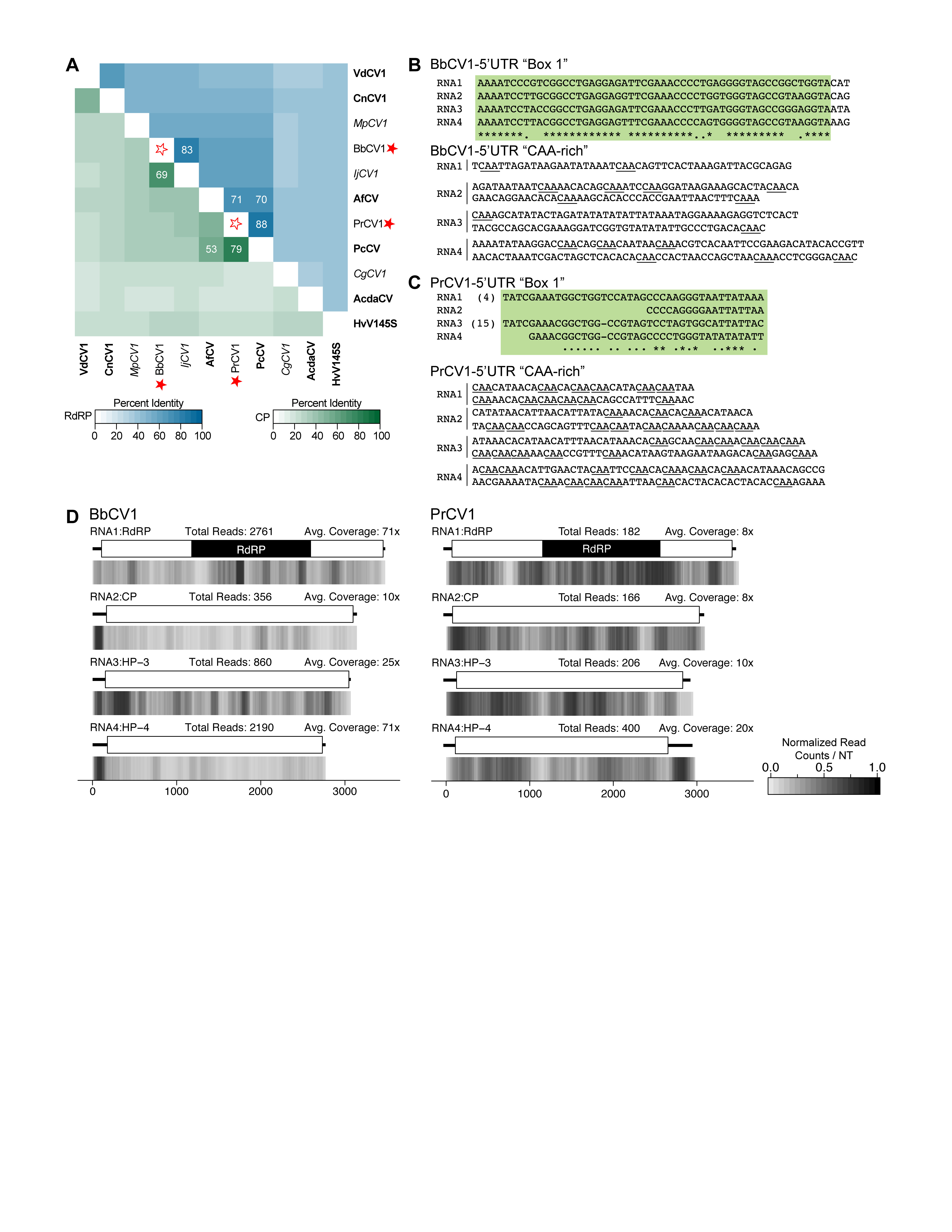

Supplement: S6 Fig — (A) Percent identity matrix, generated by Clustal-Omega 2.1, between the chrysoviruses identified in this study versus select members of the genus Chrysovirus. The top half of the matrix, in the blue scale, is the percent identity of RdRP protein sequences and the bottom half of the matrix, in the green scale, is the percent identity of CP protein sequences. For sake of clarity, the 100% identity along the diagonal has been removed and outlined stars are used to note junction point for the row and column of the viruses identified. Values above 70% for RdRP sequences and 53% for CP sequences are indicated. ICTV-member and unclassified viruses are noted in bold and italics respectively. Full names and accession numbers for protein sequences are in S2 Table. (B and C) Alignment of the 5’UTR sequences for the four genomic segments of BbCV1 (B) and PrCV1 (C). Two highly conserved regions are found in the 5’UTR; the first is a 40-75 nt region, highlighted in green, while the second region is rich in CAA repeats (underlined). (C) Density plot of read counts per nucleotide across the viral genome segments. Graphical depiction of the genome structure and predicted ORFs and motifs are on top and on the bottom is a heatmap of read counts normalized to a 0–1 scale, where the maximum read count for a given genome equals 1. Total reads mapped to the virus genome and average read coverage are noted. Red stars indicate viruses identified in this study. RdRP: RNA-dependent RNA Polymerase; CP: Coat Protein; HP: Hypothetical Protein. (TIF) [file pone.0219207.s006.tif]

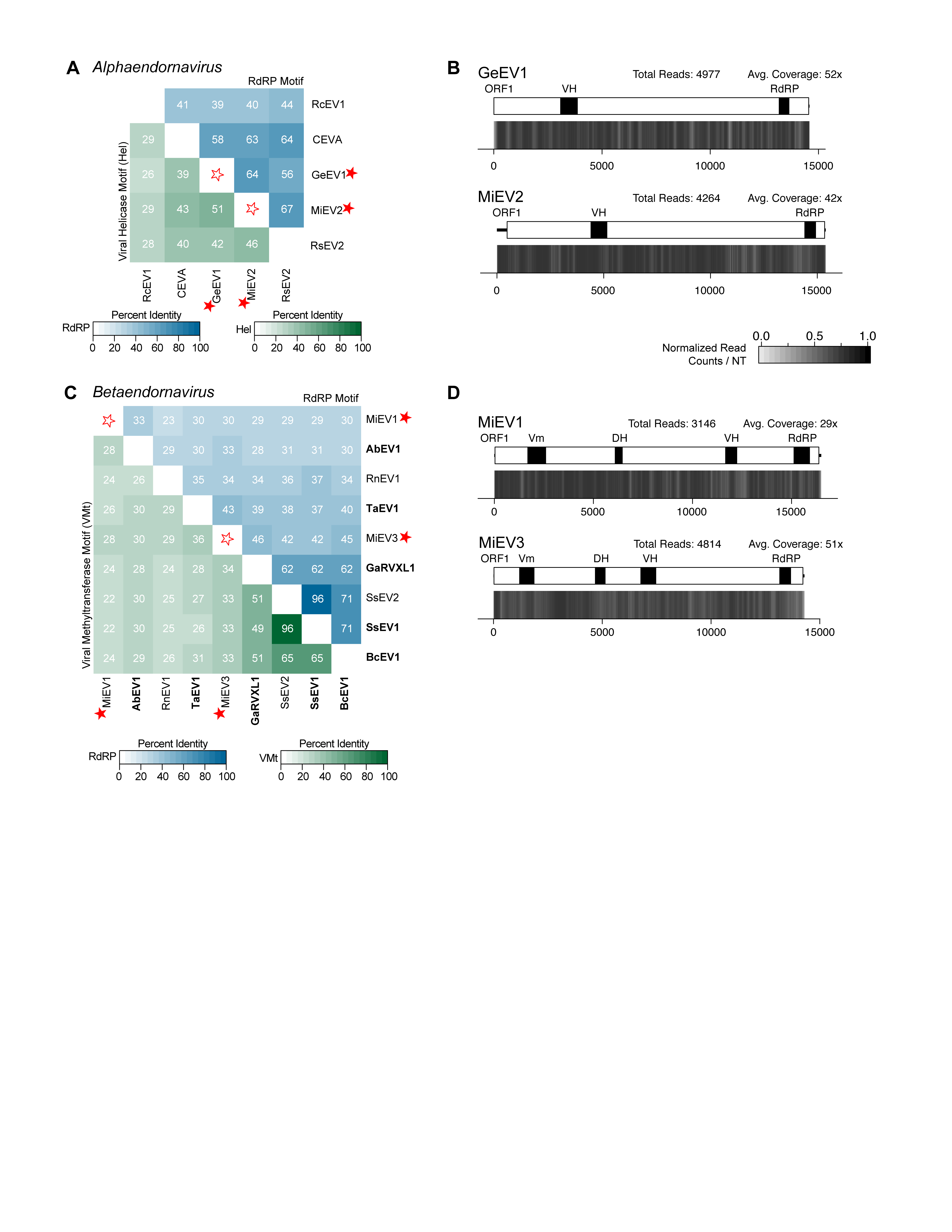

Supplement: S7 Fig — (A and C) Percent identity matrix, generated by Clustal-Omega 2.1, of mycoviruses in the genus Alphaendornavirus (A) and Betaendornavirus (C). The top half of both matrices, in the blue scale, is the percent identity of the RdRP motif sequences; the bottom half of the matrix, in the green scale, is the percent identity of the Viral Helicase 1 motif (A) and Viral methyltransferase motif (C). For sake of clarity, the 100% identity along the diagonal has been removed and outlined stars are used to note junction point for the row and column of the viruses identified in this study. Bolded names are ICTV-recognized member species. Full names and accession numbers for protein sequences are in S2 Table. (B and D) Density plot of read counts per nucleotide across the viral genome for GeEV1 and MiEV2 (B) and MiEV1 and MiEV3 (D). Graphical depiction of the genome structure, predicted ORF, and encoded motifs is on the top and a heatmap of normalized read counts is on the bottom. Read counts are normalized to a 0–1 scale, where the maximum read count for a given genome equals 1. Total reads mapped to the virus genome and average read coverage are noted. Viruses identified in this study are noted with a red star. RdRP: RNA-dependent RNA Polymerase; VH: Viral Helicase 1; Vmt/Vm: Viral methyltransferase; DH: DEXDx Helicase. (TIF) [file pone.0219207.s007.tif]

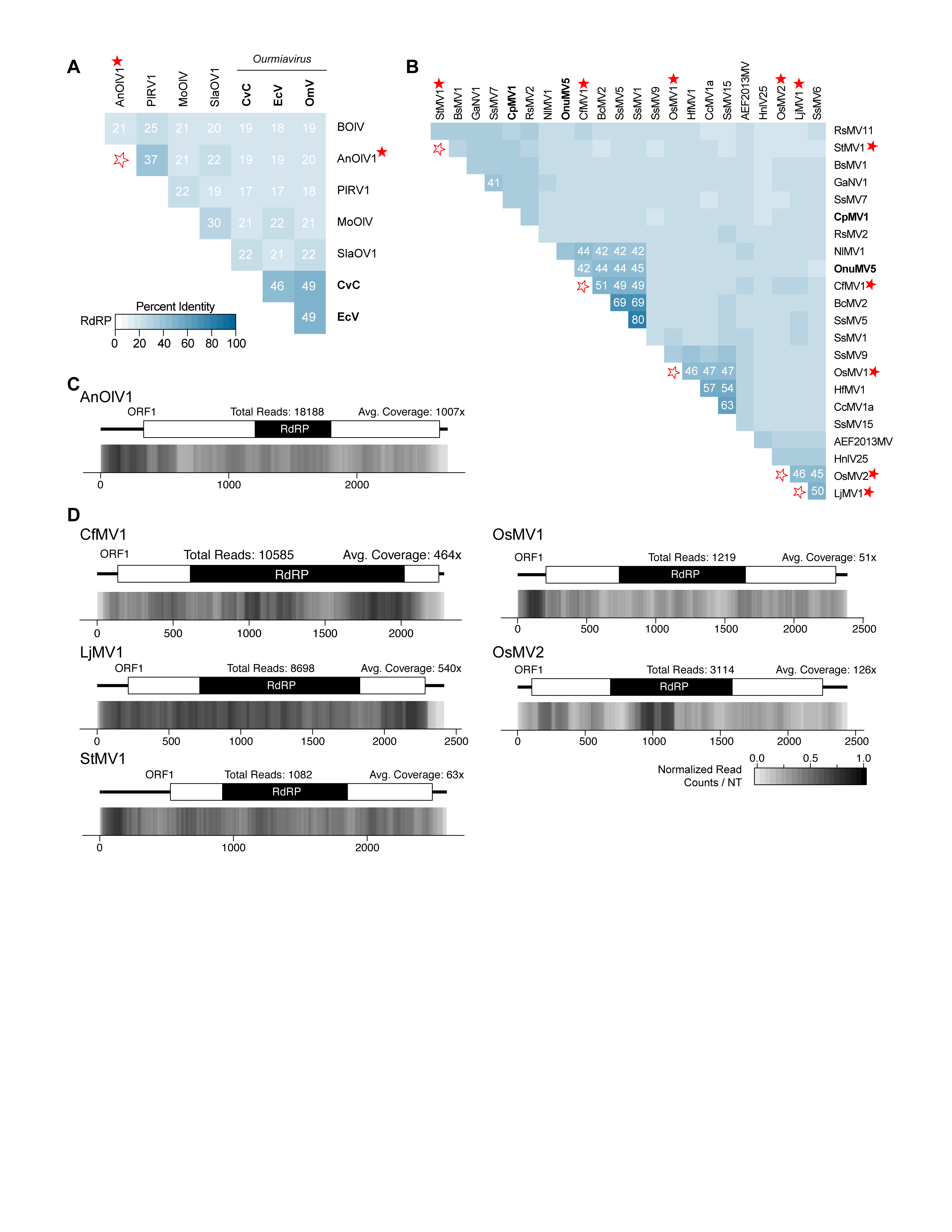

Supplement: S8 Fig — (A and B) Percent identity matrix of the RdRP protein coding sequence, generated by Clustal-Omega 2.1, of ourmia-like mycoviruses (A) and the genus Mitovirus from the family Narnaviridae (B). For the sake of clarity, only the top half of the matrix is shown and the 100% identity along the diagonal has been removed; outlined stars are used to note junction point for the row and column of the virus(es) identified. The heatmap scale in (B) is the same as found in (A). Bolded names are ICTV-recognized member species and sequences used in analysis can be found in S2 Table. (C and D) Density plot of read counts per nucleotide across the viral genomes of the ourmia-like viruses (C) and mitoviruses (D) identified in this study. Graphical depiction of the genome structure, ORF, and predicted RdRP motif for each genome are along the top and a heatmap of normalized read counts is on the bottom. Read counts are normalized to a 0–1 scale, where the maximum read count for a given genome equals 1. Total reads mapped to the virus genome and average read coverage are noted. Viruses identified in this study are noted with a red star. RdRP: RNA-dependent RNA Polymerase. (TIF) [file pone.0219207.s008.tif]

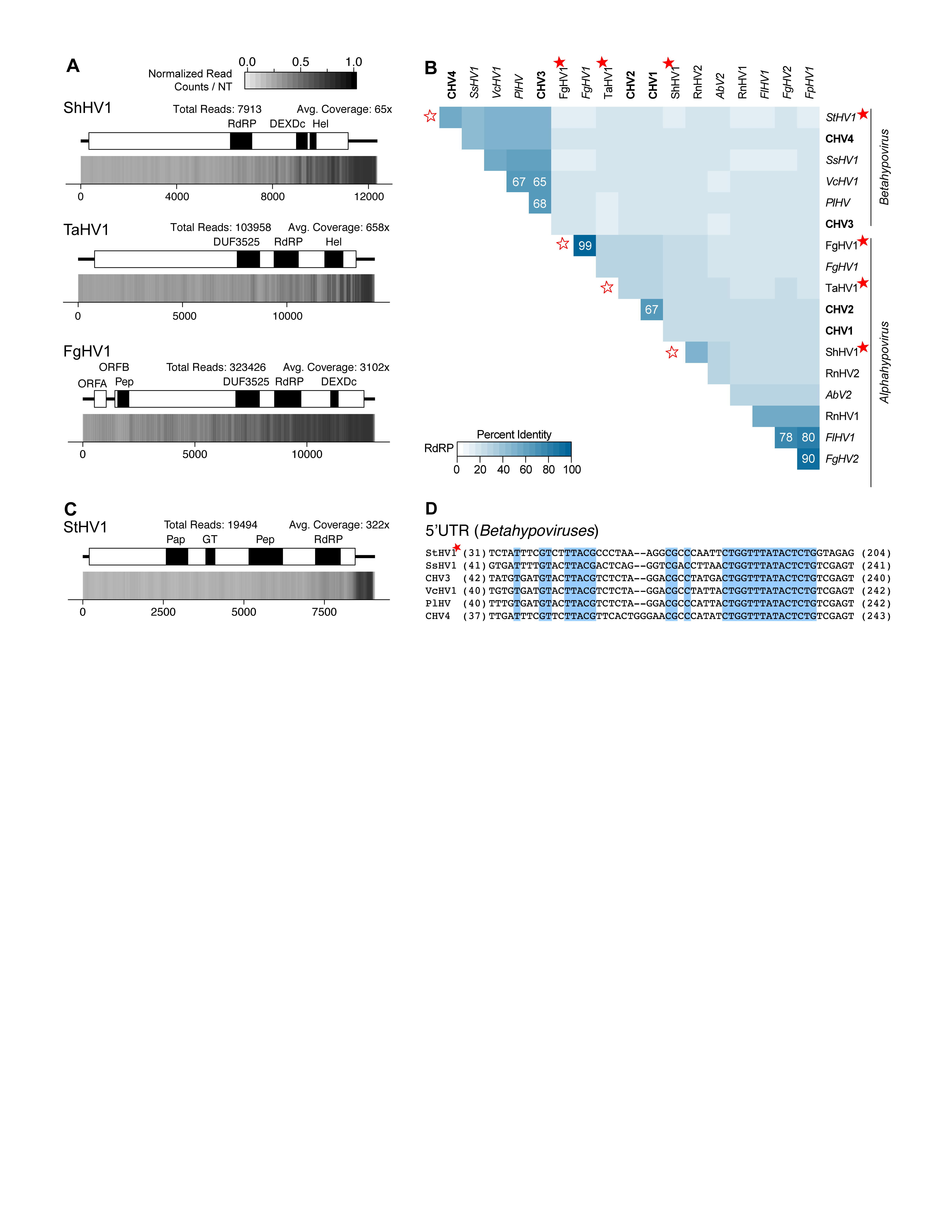

Supplement: S9 Fig — (A and C) Density plot of read counts per nucleotide across the viral genomes that group with betahypoviruses (A) and alphahypoviruses (C). Graphical depiction of the genome structure, predicted ORFs, and encoded motifs are along the top and a heatmap of normalized read counts is on the bottom. Read counts are normalized to a 0–1 scale, where the maximum read count for a given genome equals 1. Total reads mapped to the virus genome and average read coverage are noted. (B) Percent identity matrix, generated by Clustal-Omega 2.1, of the RdRP-motif containing protein coding sequence from the newly identified viruses and related hypoviruses. For sake of clarity, the 100% identity along the diagonal has been removed and outlined stars are used to note the junction point for the row and column of the viruses identified. Where sequences have similarity ≥60% is indicated. Bolded names are ICTV-recognized member species while italicized names are related, but unclassified viruses. Full names and accession numbers for sequences used are in S2 Table. (D) Alignment of 5’UTR sequences from alphahypoviruses; conserved nucleotides are highlighted in blue and residues not included in the alignment are noted in parentheses. Viruses identified in this study are noted with a red star. Pap: papain-like cysteine protease domain homologs; RdRP: RNA-dependent RNA Polymerase; Hel: Helicase; GT: UDP-glucose/sterol glucosyltransferase; Pep: peptidase; DUF: Domain of Unknown Function. (TIF) [file pone.0219207.s009.tif]

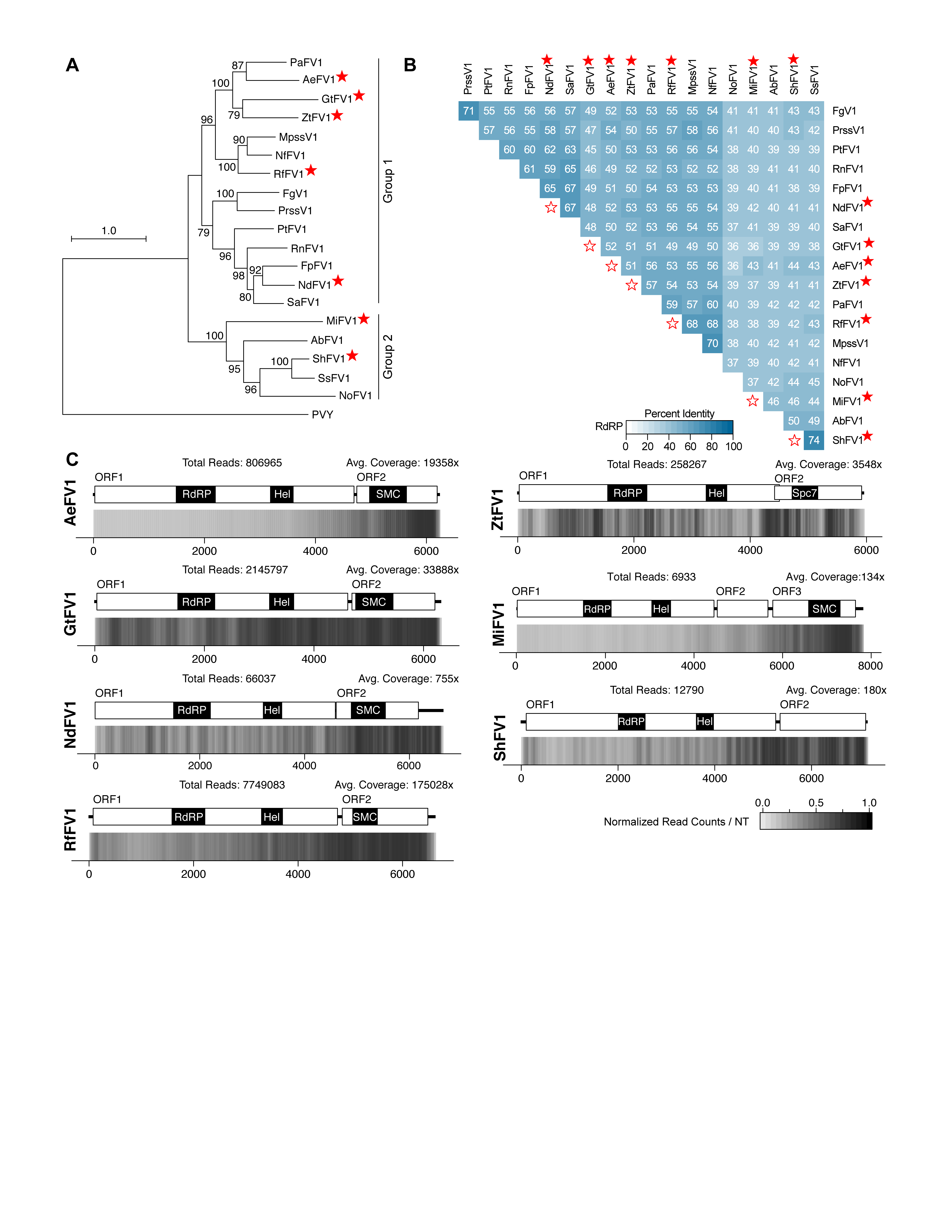

Supplement: S10 Fig — (A) Phylogram of fusariviruses based on the ORF1 protein coding sequence which contains the RdRP motif. Full names and accession numbers for protein sequences used are in S2 Table, including the outgroup Potato Virus Y (PVY). Scale bar represent 1.0 amino acid substitutions per site and numbers at the nodes indicate bootstrap support over 70% (1000 replicates). (B) Percent identity matrix generated from Clustal 2.1 using the RdRP motif sequences from fusariviruses. For sake of clarity, the 100% identity along the diagonal has been removed and open stars note the junction point between the row and column of the viruses identified in this study. (C) Density plot of read counts per nucleotide across the viral genomes identified in this study. Graphical depiction of the genome structure, predicted ORFs, and encoded motifs are on top and a heatmap of normalized read counts is on the bottom. Read counts are normalized to a 0–1 scale, where the maximum read count for a given genome equals 1. Total reads mapped to the virus genome and average read coverage are noted. RdRP: RNA-dependent RNA Polymerase; Hel: Helicase domain; SMC: SMC N terminal domain; Spc7: Spc7 kinetochore domain. (TIF) [file pone.0219207.s010.tif]

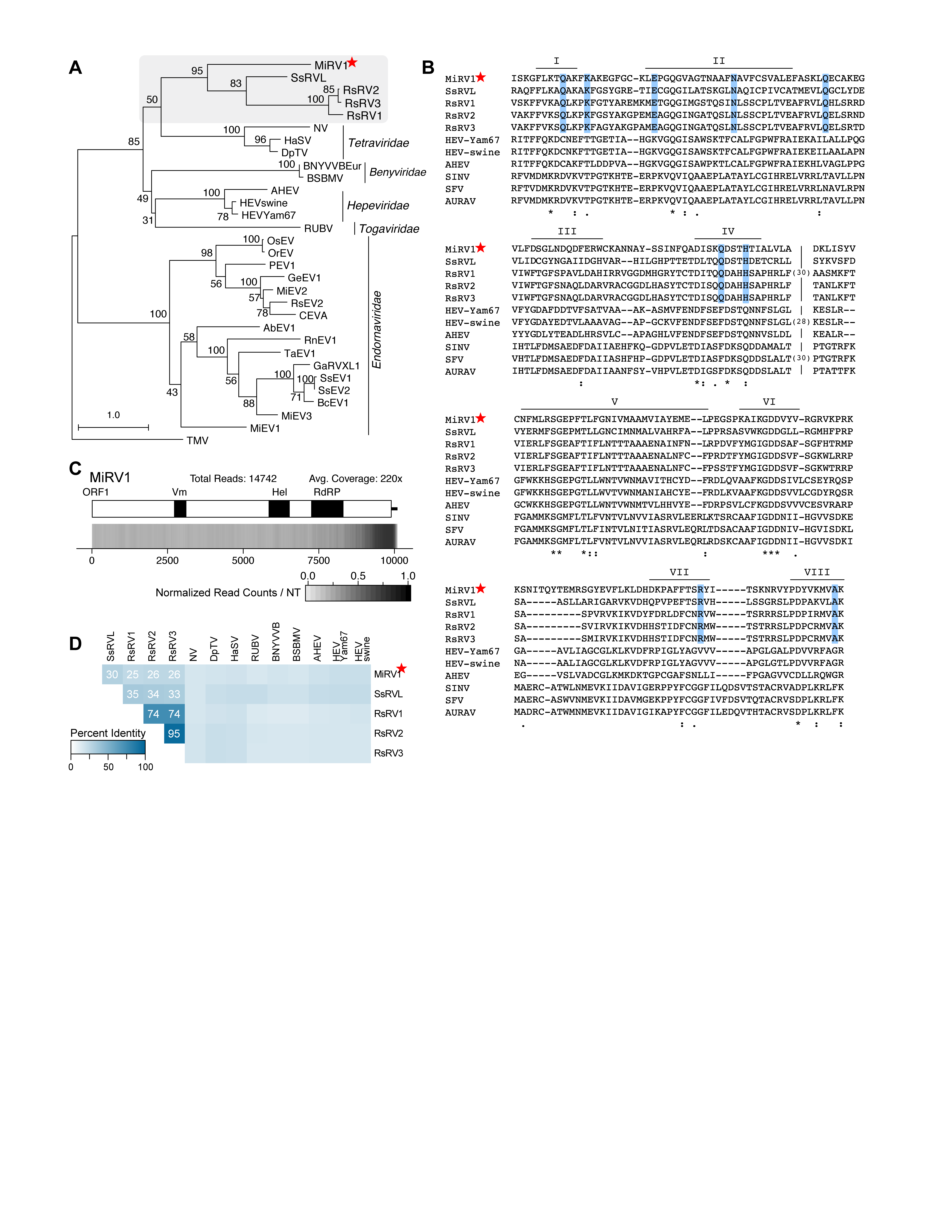

Supplement: S11 Fig — (A) Phylogram of the RdRP-motif for MiRV1, related mycoviruses, and Alpha-like viruses. Full names and accession numbers for protein sequences used are in S2 Table, including the outgroup Tobacco Mosaic Virus (TMV). Scale bar represent 1.0 amino acid substitutions per site and numbers at the nodes indicate bootstrap support over 70% (1000 replicates). (B) Alignment of the RdRP motif from MiRV1 and related sequences. Conserved motifs within (+)ssRNA RdRP sequences are noted (I to VIII) and amino acid residues found only in the five related mycoviral sequences are highlighted in blue. Numbers in brackets are amino acids not shown. (C) Density plot of read counts per nucleotide across the MiRV1 genome. Graphical depiction of the genome structure, predicted ORF, and encoded motifs are along the top, and a heatmap of normalized read counts is on the bottom. Read counts are normalized to a 0–1 scale, where the maximum read count equals 1. Total reads mapped to the virus genome and the average read coverage are noted. (D) Percent identity matrix generated from Clustal 2.1 using the RdRP motif sequences from MiRV1, mycoviral relatives and select Alpha-like viruses. For sake of clarity, the 100% identity along the diagonal has been removed and only identities ≥ 25% are indicated. Red star notes MiRV1, the virus identified in this study. RdRP: RNA-dependent RNA Polymerase; Vm: Viral methyltransferase; Hel: Helicase. (TIF) [file pone.0219207.s011.tif]
